# *microRNA-200c* Mitigates Pulpitis and Promotes Dentin Regeneration

**DOI:** 10.3390/ijms26146734

**Published:** 2025-07-14

**Authors:** Tadkamol Krongbaramee, Chawin Upara, Matthew T. Remy, Long Jiang, Jue Hu, Kittiphoj Tikkhanarak, Bruno Cavalcanti, Hongli Sun, Fabricio B. Teixeira, Liu Hong

**Affiliations:** 1 Iowa Institute for Oral Health Research, College of Dentistry, the University of Iowa, Iowa City, IA 52242, USA; tadkamol.k@gmail.com (T.K.); chawin-upara@uiowa.edu (C.U.); matt-remy@uiowa.edu (M.T.R.); jianglong25a@163.com (L.J.); jue-hu@uiowa.edu (J.H.); hongli-sun@uiowa.edu (H.S.); 2 Division of Endodontics, Department of Restorative Dentistry & Periodontology, Faculty of Dentistry, Chiang Mai University, Chiang Mai 50200, Thailand; 3 Department of Oral Pathology, Radiology, and Medicine, College of Dentistry, the University of Iowa, Iowa City, IA 52242, USA; kittiphoj-tikkhanarak@uiowa.edu; 4 Department of Cariology, Restorative Sciences and Endodontics, School of Dentistry, University of Michigan, Ann Arbor, MI 48109, USA; brunocav@umich.edu; 5 Department of Endodontics, College of Dentistry, the University of Iowa, Iowa City, IA 52242, USA; fabricio-teixeira@uiowa.edu

**Keywords:** *miR-200c*, pulpitis, proinflammatory cytokine, odontogenic differentiation, nanoparticles

## Abstract

*MicroRNA (miR)-200c* enhances osteogenesis, modulates inflammation, and participates in dentin development. This study was to investigate the beneficial potential of *miR-200c* in vital pulp therapy (VPT) by mitigating pulpitis and promoting dentin regeneration. We explored the *miR-200c* variations in inflamed pulp tissues from patients with symptomatic irreversible pulpitis and primary human dental pulp-derived cells (DPCs) challenged with *P.g.* lipopolysaccharide (*Pg*-LPS). We further assessed the functions of overexpression of *miR-200c* on odontogenic differentiation, pulpal inflammation, and dentin regeneration in vitro and in vivo. Our findings revealed a noteworthy downregulation of *miR-200c* expression in inflamed pulp tissues and primary human DPCs. Through the overexpression of *miR-200c* via transfecting plasmid DNA (pDNA), we observed a substantial downregulation of proinflammatory cytokines interleukin (IL)-6 and IL-8 in human DPCs. Furthermore, this overexpression significantly enhanced the transcript and protein levels of odontogenic differentiation markers, including Runt-related transcription factor (Runx)2, osteocalcin (OCN), dentin matrix protein (DMP)1, and dentin sialophosphoprotein (DSPP). In a rat model of pulpitis induced by *Pg*-LPS, we demonstrated notable benefits by local application of pDNA encoding *miR-200c* delivered by CaCO_3_-based nanoparticles to reduce pulpal inflammation and promote dentin formation. These results underscore the significant impact of locally applied *miR-200c* in modulating pulpal inflammation and facilitating dentin repair, showcasing its ability to improve VPT outcomes.

## 1. Introduction

Pulpitis is a progressive infection and inflammation of the dentin–pulp complex, resulting in increased pulp chamber’s internal pressure, pulp tissue ischemia, and severe pain [1]. A pulpectomy is the traditional therapy of removing the pulp tissue to eliminate pulpal infection, inflammation, and pain. However, patients cannot perceive pain in the tooth after a pulpectomy procedure, and as a result, the tooth becomes fragile and prone to root fracture due to a lack of resistance to infection and disease [1]. Vital pulp therapy (VPT), including partial pulpotomy and direct pulp capping, is an alternative treatment that potentially averts side effects of traditional pulpectomy and improves outcomes of irreversible pulpitis treatment [2,3,4,5,6]. However, VPT remains disappointing in permanent teeth of older patients, and the treatment outcomes significantly decline over time [7,8,9]. The success rate of calcium silicate-based material for partial and pulpotomy, including mineral trioxide aggregate (MTA), the most advanced material in VPT, remains similar to currently used calcium hydroxide [10]. MTA also has limitations, such as incomplete reparative hard tissue formation, complex handling properties, and tooth discoloration [11,12,13].

While mild inflammation enhances odontogenic differentiation and dentin repair, excessive inflammation worsens pulpitis recovery [14,15]. Upon binding to Toll-like receptors, NOD-like receptors, and inflammasomes in pulp cells, bacterial ligands activate proinflammatory signaling pathways, leading to amplified secretion of extracellular cytokines, chemokines, and inflammatory molecular mediators. Consequently, these cytokines and mediators recruit and activate immune system cells, releasing reactive oxygen species and potent enzyme proteases, ultimately causing substantial pulp cell death and tissue damage [16]. Therefore, mitigating inflammation is imperative to protect the pulp-dentin complex from irreversible damage and foster odontogenic differentiation [3,17].

MicroRNA (miRNAs) regulate signal pathways in physiology and pathophysiology, including tooth development [18,19] and pulpitis [20,21,22,23]. Specific miRNAs vary in inflamed human pulp tissues and dental pulp stem cells (DPSCs) under lipopolysaccharide (LPS) stimulation and exhibit strong modulating inflammation capabilities [21,22,23,24,25]. Specifically, *miR-410, -30b,* and *-204* were downregulated in inflamed human pulp tissues, and overexpression of *miR-146a* and *-506* and downregulation of *miR-21* were found in DPSCs stimulated using LPS [21,22,23,24,25]. Modifying the expression levels of *let-7c-5p*, *miR-146,* -*497-5p*, *-143-3p*, *-508-5p*, *-675*, and *-34a*, either by upregulation or downregulation, has been reported to promote odontogenic differentiation of DPSCs in vitro. Local application of *let-7c-5p* attenuated LPS-induced pulpitis in a rat model by inhibiting the dentin matrix acidic phosphoprotein (DMP)1-mediated nuclear factor kappa-light-chain-enhancer of activated B cells (NF-κB) pathway [26,27,28,29,30]. Notably, *miR-200c* plays a crucial role in dentin development of Wnt and BMP (Bone Morphogenetic Protein) signaling by targeting noggin [18]. It also enhances osteogenesis by targeting SRY-related HMG-box 2 (SOX2) and Krüppel-like factor 4 (KLF4) [31] in Wnt signaling and myeloid differentiation primary response 88 (MYD88) in AKT-β-catenin signaling [32]. Moreover, *miR-200c* demonstrates the ability to modulate inflammation by reducing NF-κB activation through Toll-like receptor 4 (TLR4) and MyD88-dependent pathways [33] and targeting inhibitor of nuclear factor kappa B kinase subunit beta (IKBKB) in the NF-κB signal pathway [34]. Previous studies have further highlighted the direct targeting capabilities of *miR-200c* on interleukin (IL)-6, IL-8, and C-C motif chemokine ligand 5 (CCL-5), resulting in the effective downregulation of these proinflammatory mediators and attenuation of inflammation [35,36,37].

The objectives of the present study were to explore variations in *miR-200c* in inflamed human pulp tissues and primary human dental pulp-derived cells (hDPCs). We aimed to show that transfecting plasmid DNA (pDNA) encoding *miR-200c* can lead to anti-inflammatory effects and enhance odontogenic differentiation and mineralization in hDPCs and human dental pulp stem cells (DPSCs). Additionally, the project sought to validate whether pDNA *miR-200c* effectively reduces inflammation and whether its delivery using biocompatible CaCO_3_-based nanoparticles can promote dentin regeneration in rat models of pulpitis. Ultimately, this project aimed to demonstrate the feasibility of locally administering *miR-200c* to improve the current clinical outcomes of vital pulp therapy (VPT).

## 2. Results

### 2.1. miR-200c Participates in the Pathogenesis of Pulpitis and Inflamed Human Dental Pulp Cells (hDPCs)

**Figure 1:**
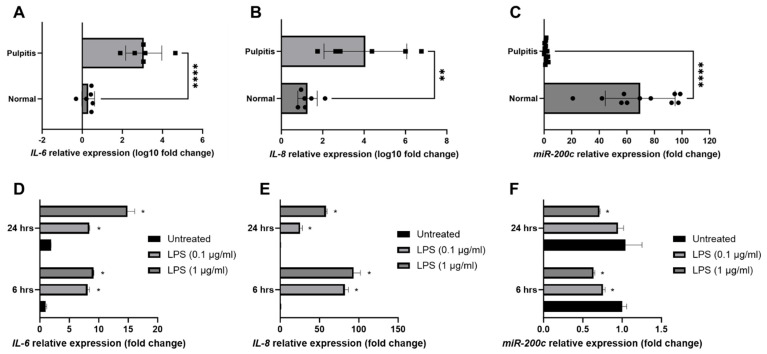
*miR-200c* was downregulated in inflamed human pulp tissues and cells. (**A**–**C**) Normalized log10 fold changes in *IL-6* (**A**), *IL-8* (**B**), and *miR-200c* (**C**) transcript in pulp tissues of irreversible pulpitis, compared to the healthy pulp. *n* = 6–11, *: *p* < 0.05, **: *p* < 0.01, ****: *p* < 0.001 vs. healthy controls; (**D**–**F**) Normalized fold changes in *IL-6* (**D**), *IL-8* (**E**), and *miR-200c* (**F**) transcripts in human dental pulp cells 6 and 24 h after stimulation with *Pg*-LPS at 0.1 and 1 µg/mL. *: *p* < 0.05 vs. untreated; *n* = 3.

To investigate the participation of *miR-200c* in pulpitis patients, the inflamed pulp tissues were extirpated from carious teeth diagnosed with irreversible pulpitis patients (*n* = 11) according to the American Association of Endodontists guidelines at the University of Iowa College of Dentistry and Dental Clinics. The healthy pulp controls (*n* = 11) were extirpated from healthy third molars with complete root formation or teeth extracted during orthodontic treatment. Total RNA was extracted from the pulp tissues and quantitatively analyzed using qRT-PCR. The transcripts of *IL-6* and *IL-8* increased intensely in inflamed tissues relative to healthy pulps (Figure 1A,B, *n* = 6). Notably, *miR-200c* expression was significantly reduced in inflamed pulp (Figure 1C, *n* = 11). To quantify the amount of inflammation and the *miR-200c* variations in inflamed hDPCs, the primary hDPCs were extracted from healthy third molars. After hDPCs were treated with *P. gingivalis* lipopolysaccharide (*Pg*-LPS) at 0.1 and 1 µg/mL to induce inflammation, the transcripts of *IL-6* and *IL-8* were significantly increased (Figure 1D,E, *n* = 3). Notably, *miR-200c* expression was significantly reduced in inflamed hDPCs (Figure 1F, *n* = 3).

### 2.2. miR-200c Overexpression Promotes Odontogenic Differentiation in Human Dental Pulp Stem Cells (DPSCs) and Mitigates IL-6 and IL-8 in Inflamed Human Dental Pulp Cells (hDPCs)

**Figure 2:**
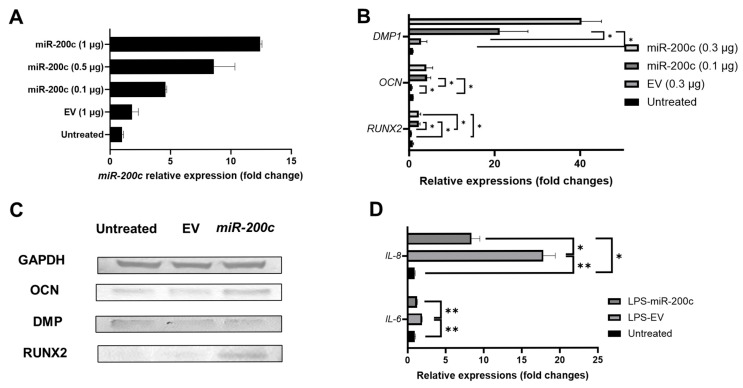
*miR-200c* promoted odontogenic differentiation of human DPSCs and reduced IL-6 and IL-8 in human DPCs. (**A**) *miR-200c* transcript in DPSCs treated with pDNA *miR-200c* at different doses; *n* = 2. (**B**) Normalized fold changes in DMP1, OCN, and Runx2 in human DPSCs 7 days after treatment with *miR-200c* and controls. *: *p* < 0.05; *n* = 3. (**C**) Western blot of DMP1, OCN, and Runx2 in human DPSCs 14 days after treatment with 0.1 µg *miR-200c* and controls. (**D**) *IL-6* and *IL-8* transcript in human DPCs pretreated with 0.1 µg pDNA *miR-200c* or EV 6 h after challenged with *Pg*-LPS. *: *p* < 0.05, **: *p* < 0.01; *n* = 3.

To evaluate the function of *miR-200c* on odontogenesis and inflammation in vitro, pDNA *miR-200c* was transfected to human DPSCs and hDPCs using polyethyleneimine (PEI)-based nanoparticles. The increased *miR-200c* expression was confirmed in a dose-dependent manner (Figure 2A, *n* = 3). *miR-200c* treatment at 0.1 and 0.3 μg significantly increased the transcripts of *runt-related transcription factor 2* (*Runx2*), *osteocalcin* (*OCN*), and *DMP1* compared to untreated control and treatment with empty vector (EV) after seven days (Figure 2B, *n* = 3). These odontogenic differentiation markers were also improved after 14 days, as confirmed by Western blot (Figure 2C, see quantitative analysis at the Supplementary Figures S1–S3). Additionally, the hDPCs transfected with EV significantly increased transcripts of *IL-6* and *IL-8* after challenging with *Pg*-LPS. However, pretreatment with pDNA *miR-200c* significantly downregulated *IL-6* and *IL-8* transcripts (Figure 2D, *n* = 3).

### 2.3. Biocompatible CaCO3-Based Nanoparticles Significantly Improve the Transfection Efficiency of miR-200c in Dental Pulp Stem Cells

**Figure 3:**
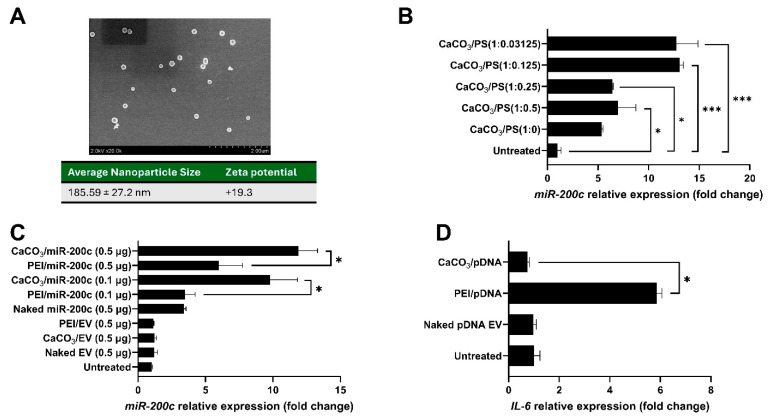
Biocompatible CaCO_3_ nanoparticles improved the transfection efficiency of *miR-200c*. (**A**) SEM images and characteristics of CaCO_3_/*miR-200c* nanocomplexes. (**B**) Normalized transcript of *miR-200c* delivered by CaCO_3_ nanoparticles with different ratios of PS. *: *p* < 0.05. ***: *p* < 0.005, *n* = 3. (**C**,**D**) Normalized transcripts of *miR-200c* (**C**) and *IL-6* (**D**) in DPSCs treated with pDNA *miR-200c* with different delivery systems. *: *p* < 0.05. *n* = 3.

The CaCO_3_/pDNA *miR-200c* nano-complexes were freshly prepared using a previously reported method [38,39,40]. As illustrated in Figure 3, the diameters of CaCO_3_/*miR-200c* nanocomplexes at CaCO_3_: Protamine sulfate (PS) of 1:0.25 were 185.59 ± 27.2 nm, and their Zeta potential was +19.3 (Figure 3A). Interestingly, the CaCO_3_/PS ratio impacts transfection efficiencies of pDNA encoding *miR-200c*, and the expression of *miR-200c* was upregulated as PS concentrations increased. CaCO_3_/PS at ratios of 1:0.25 and 1:0.5 showed the best transfection efficiencies (Figure 3B, *n* = 3). PEI and CaCO_3_ significantly increased the *miR-200c* expression than naked pDNA *miR-200c*. However, CaCO_3_ transfected significantly higher amounts of *miR-200c* than did PEI (Figure 3C, *n* = 3). Notably, the delivery using PEI upregulated *IL-6* transcripts; however, the delivery using CaCO_3_-based nanoparticles has significantly lower *IL-6* expression (Figure 3D, *n* = 3).

### 2.4. miR-200c Delivered by CaCO_3_ Promotes Odontogenic Differentiation in Human DPSCs

**Figure 4:**
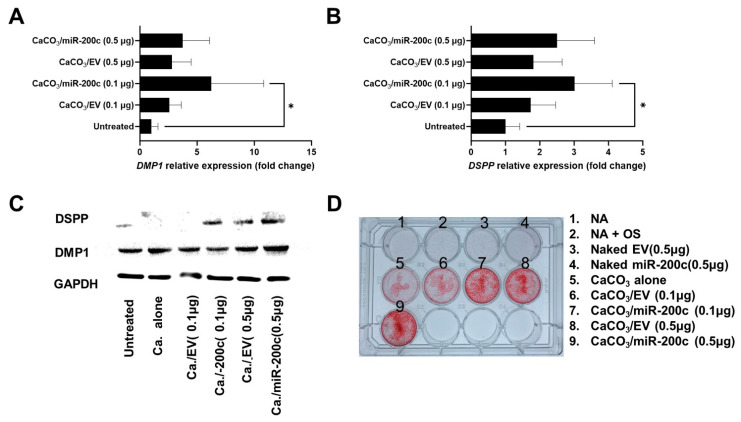
*miR-200c* delivered by CaCO_3_-based nanoparticles reduced proinflammatory cytokines and promoted odontogenic differentiation in vitro. (**A**,**B**) Normalized transcripts of DMP1 (**A**) and DSPP (**B**) of human DPSCs pretreated with CaCO_3_/*miR-200c* at 0.1 and 0.5 µg 12 days after cultured in odontogenic support medium. *: *p* < 0.05; *n* = 3. (**C**,**D**) The images of Western blot for DMP1 and DSPP (**C**) and Alizarin Red staining (**D**) of DPSCs with different treatments of CaCO_3_/*miR-200c*. NA: non-transfected. NA + OS: non-transfected cells with osteogenic medium.

As illustrated in Figure 4, transfection of pDNA *miR-200c* using CaCO_3_ significantly increased transcripts of DMP1 (Figure 4A, *n* = 3) and dentin sialophosphoprotein (DSPP) (Figure 4B, *n* = 3). The Western blot showed that the band intensities of DSPP and DMP1 were increased in DPSCs treated with *miR-200c* at different concentrations (Figure 4C, see quantitative analysis at the Supplementary Figures S1–S3). In addition, Alizarin Red staining demonstrated that pDNA *miR-200c* delivered by CaCO_3_-based nanoparticles improved the mineralization in DPSCs more than EV and naked pDNA *miR-200c* (Figure 4D).

### 2.5. miR-200c Mitigates Pulp Inflammation and Improves Dentin Regeneration In Vivo

**Figure 5:**
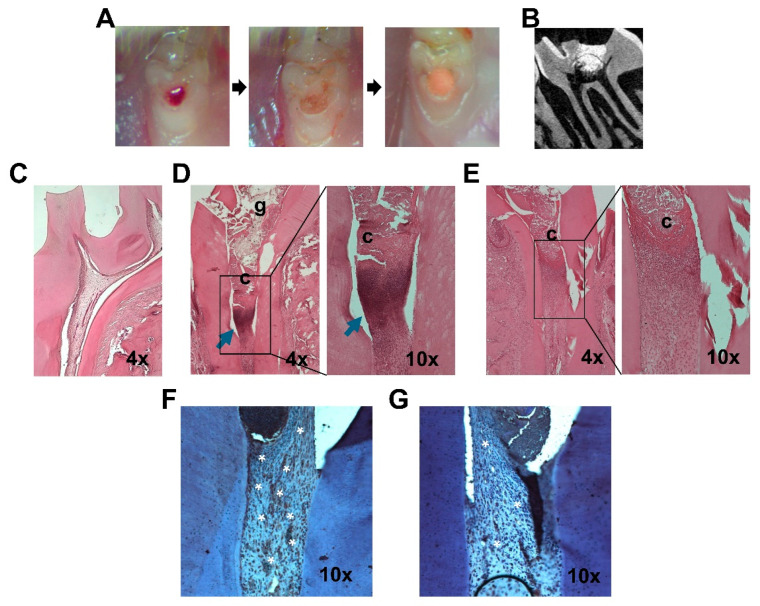
Histological examination of *miR-200c* mitigating inflammation in a rat model of pulpitis. (**A**) The surgical procedure images to create a rat model of by *Pg*-LPS-induced pulpitis. (**B**) A representative cross-section µCT image of a rat model of pulpitis one week post-operatively. (**C**) A cross-section of healthy rat pulp tissues stained with H&E. (**D**,**E**) Representative cross-sections of pulp tissues 7 days after treatment with collagen sponges soaking pDNA *miR-200c* (**E**) and EV (**D**) on *Pg*-LPS-induced rat pulpitis stained with H&E. c: collagen sponge, g: glass ionomer, arrows: infiltration of inflammatory cells. (**F**,**G**) Representative cross-sections of pulp tissues treated with collagen sponges incorporating pDNA *miR-200c* (**G**) and EV (**F**) on *Pg*-LPS-induced rat pulpitis stained with IHC with anti-IL-6 antibodies, *: positive IL-6 staining.

A modified rat model of pulpitis was used to determine the functions of *miR-200c* in pulp inflammation and dentin regeneration [17,41] (Figure 5A,B). Compared to healthy dental pulp tissues (Figure 5C), the typical inflammation in rat pulpal tissues challenged with *Pg*-LPS was observed in a rat model while treated with EV after seven days, presenting a greater density of inflammatory infiltrates in the connective tissue underneath the restorative material (blue arrowed) (Figure 5D). However, the *miR-200c* treatment effectively reduced inflammatory cell infiltration (Figure 5E), showing that a significant decrease in inflammatory cell infiltration was observed in pulp tissue pretreated with *Pg*-LPS and capping with *miR-200c* incorporated collagen sponge. Additionally, the IHC staining using an anti-IL-6 antibody indicated that treatment with *miR-200c* effectively reduced IL-6 (stained in brown color) in the pulpal tissues induced by *Pg*-LPS more so than EV (Figure 5F,G).

**Figure 6:**
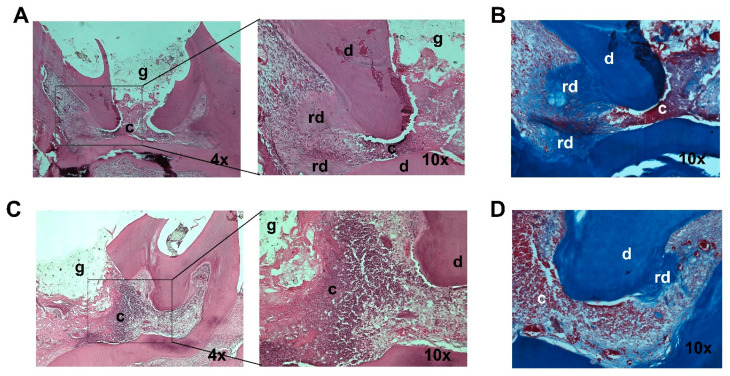
Histological examination of *miR-200c* improving dentin regeneration in a rat model of pulpitis. Representative cross-section images of pulp tissue 3 weeks after treatment with collagen sponges incorporating CaCO3/pDNA *miR-200c* (**A**,**B**) and CaCO_3_/EV at LPS-induced pulpitis of rats (**C**,**D**). Stained with H&E (**A**,**C**) and Heidenhain’s AZAN (**B**,**D**). c: collagen sponge, g: Glass ionomer, d: dentin, rd: reparative dentin. *n* = 3.

To determine the function of *miR-200c* delivered by CaCO_3_ nanoparticles in mitigating inflammation and promoting dentin formation in a rat model of pulpitis. CaCO_3_/*miR-200c* at a ratio of CaCO_3_/PS at 1:0.25 were incorporated into trimmed collagen sponges. After rat pulpitis was created at the maxillary first molars by adding *Pg*-LPS into the cavity, the injured site was then covered with the collage sponge incorporated with Ca-CO_3_/*miR-200c*. After 3 weeks, we found new tissue formation consistent with reparative dentin adjacent to collagen with CaCO_3_/*miR-200c* (Figure 6A,B). However, the treatment with CaCO_3_/EV has limited dentin formation (Figure 6C,D). To assess the severity of inflammation reduced by *miR-200c*, we scored pulp inflammation under various treatment conditions in a double-blinded manner [42]. The scores for the inflammatory cell response in pulpitis treated with *miR-200c* were generally lower than those treated with EV (refer to the Supplementary Figures S1–S3).

## 3. Discussion

This study marks a pioneering effort to demonstrate the feasibility of using miRNA-based gene therapy delivered via biocompatible non-viral nanoparticles for potential clinical application in attenuating inflammation and simultaneously promoting odontogenesis and dentin regeneration. Our investigation revealed the downregulation of *miR-200c* in irreversible pulpitis patient pulp tissues and inflamed human pulp cells. Through the transfection of pDNA encoding *miR-200c* using nanoparticles, the overexpression of *miR-200c* exhibited potent anti-inflammatory effects and enhanced odontogenic differentiation and dentin formation in human DPSCs and preclinical models of pulpitis. These findings strongly indicate that local application of pDNA encoding *miR-200c* has the potential to improve the outcomes of current VPT by mitigating inflammation and promoting odontogenesis.

In this study, pDNA encoding *miR-200c* was utilized instead of chemically synthesized miRNA mimics. This approach mimics the biogenesis of endogenous miRNAs, circumventing the accumulation of high molecular weight RNA species that may occur with the application of miRNA mimics [43]. Furthermore, using pDNA encoding miRNAs allows for the sustained maintenance of a relatively high concentration, optimizing its efficacy in modulating inflammation and odontogenesis. This contrasts with the potential for supraphysiological levels of mature miRNAs induced by mimics, which may lead to non-specific changes in gene expression [44,45]. The application of biocompatible and biodegradable CaCO_3_-based nanoparticles has been demonstrated to enhance the transfection efficiencies of pDNA in gene therapy. Previous studies have demonstrated the effective transfection of pDNA encoding *miR-200c* using CaCO_3_-based nanoparticles in contexts such as bone regeneration and anti-oral squamous cell carcinoma [39,40]. In this study, we further established that CaCO_3_ effectively improves the transfection efficiency of pDNA *miR-200c* into DPSCs with less toxicity than PEI. Collectively, this evidence supports the conclusion that pDNA-*miR-200c* delivered by CaCO_3_-based nanoparticles effectively enhances the functionality of *miR-200c* in regulating inflammation and promoting odontogenic differentiation.

Regarding potential mechanism(s) associated with *miR-200c* in dentin regeneration and pulpitis mitigation, prior studies have demonstrated that *miR-200c* exhibits the remarkable ability to target and inhibit the BMP antagonist noggin, a crucial factor known for its role in upregulating endogenous BMP signaling activities in dentin development [18]. Moreover, previous studies have demonstrated that *miR-200c* can effectively enhance Wnt/β-catenin activities by targeting Sox2 and Klf4 in osteogenic differentiation and mineralization [31]. This is noteworthy as these pathways align with the signaling mechanisms involved in odontogenic differentiation and dentin regeneration. Consequently, the existing robust evidence strongly suggests that *miR-200c* promotes odontogenic differentiation and dentin regeneration, potentially by upregulating BMP and Wnt signaling through the targeted inhibition of noggin, Sox2, and KLF4. Additionally, *miR-200c* demonstrates potent anti-inflammatory activities in the context of periodontitis and cancer inflammation [33,39,40,46,47,48]. Earlier studies have also illustrated the effectiveness of *miR-200c* in mitigating inflammation by targeting IL-6 and IL-8 and downregulating NF-κB signaling, elucidating the mechanism(s) underlying the overexpression of *miR-200c* in inhibiting inflammation in pulpitis [33,35]. Furthermore, these factors and *miR-200c* appear to mutually influence each other, suggesting that the observed downregulation of *miR-200c* in the pulpitis pulp tissue and inflamed dental pulp cells may be attributed to the upregulation of noggin and proinflammatory cytokines.

In summary, this study provides compelling evidence for the role of *miR-200c* in the pathogenesis of human pulpitis and highlights its therapeutic potential in improving current vital pulp therapy (VPT) outcomes. Specifically, *miR-200c* appears to reduce inflammation and promote odontogenic differentiation, ultimately leading to dentin regeneration. However, there are several limitations that need to be addressed in future research before this approach can be implemented into clinical settings. First, it is crucial to validate the effectiveness of *miR-200c* in promoting odontogenic differentiation in aged DPSCs and in reducing inflammation in older hosts. Additionally, a thorough investigation of potential off-target effects, both locally and systemically, is essential. Moreover, we acknowledge limitations in our study. The rat tooth model with *Pg*-LPS-induced pulpitis does not accurately represent clinical human pulpitis due to differences in physiological characteristics and the pathogens involved. Furthermore, the small sample size of the rats in our study restricted our ability to perform a solid quantitative analysis, which may impact the reliability of the animal model’s outcomes. We are committed to addressing these limitations in our future research.

## 4. Materials and Methods

### 4.1. Characterizing miR-200c and Proinflammatory Cytokines Within Inflamed Human Pulp Tissues and Dental Pulp Cells

This study was approved by the Institutional Review Board at our University (IRB#201710788). According to the American Association of Endodontists guidelines, the inflamed pulp tissues were harvested from carious teeth diagnosed with irreversible pulpitis (*n* = 11). The inclusion criteria are patients who are older than 14 years old and have American Association of Anesthesiologist status I or II: ASA I: A normal healthy patient, non-smoking, no, or minimal use of alcohol; ASA II: A patient with mild systemic disease only without substantive functional limitations. Examples include but are not limited to current smokers, social alcohol drinkers, and patients with well-controlled diabetes mellitus, hypertension, or mild lung disease. Patients who are immunocompromised or have long-term use of drugs that influence the immune system were excluded. After the root chamber was accessed, the pulps were extirpated using a sterilized barbed broach or hand file. The pulp tissue was subsequently placed into Qiazol and stored at −80 °C until RNA extraction. For healthy controls, the pulp was harvested from healthy third molars with complete root formation or teeth extracted for orthodontic purposes (*n* = 11). After extraction, a 1 mm depth cut using a diamond disc along the cementoenamel junction (CEJ) was made and the pulp tissue was exposed and collected using a chisel. Transcripts of proinflammatory cytokines, including *IL-6* and *IL-8*, and *miR-200c* from the patient pulp tissues were quantified using quantitative reverse transcription PCR (qRT-PCR) and compared to healthy controls.

Human DPCs were extracted from pulp tissues of healthy third molars extracted for orthodontic purposes. The unharmed pulp tissue was minced and digested in 3.0 mg/mL collagenase type I and then cultured in a dental pulp stem cell basal medium (Lonza, Visp, Switzerland). The cells at 10^5^/per well were treated with *Porphyromonas gingivalis* lipopolysaccharide (*Pg*-LPS) (Biological Laboratories, Campbell, CA, USA) at 0.1 and 1.0 µg/mL with reduced serum medium to induce inflammation in the DPCs. The transcripts of proinflammatory cytokines and *miR-200c* were quantified using qRT-PCR.

### 4.2. Determining Odontogenic Differentiation Enhancement and Anti-Inflammation of miR-200c Overexpression in Human Dental Pulp Stem Cells and Dental Pulp Cells

Human DPSCs were purchased (PT5025, Lonza, Walkersville, MD, USA) and cultured with the DPSC BulletKit^®^ Medium (PT-3005, Lonza, Walkersville, MD, USA). The DPSC Dental Pulp Stem Cell BulletKit^®^ Medium includes both the basal media and the necessary supplements for the proliferation of human DPSC. The cells were cultured until they reached approximately 70% confluence, and then they were split into a 1:6 ratio for subculture. The human DPSC express CD105, CD166, CD29, CD90, and CD73, and to not express CD34, CD45, and CD133. pDNA encoding *miR-200c* and empty vector (EV) were prepared as described previously [31] and transfected into human DPSCs and DPCs using polyethyleneimine-based nanoparticles (PEI, MW 25 kDa; Sigma-Aldrich, St. Louis, MO, USA) [35]. Specifically, DPSCs at 10^5^ cells/well were treated with 0.1, 0.5, 1, and 5 μg of PEI/pDNA *miR-200c* nanocomplexes in Opti-MEM (Life technologies, Paisley, UK). The *miR-200c* levels were quantified after three days using qRT-PCR.

To determine the function of *miR-200c* overexpression on odontogenic differentiation, DPSCs 3 days after transfected with pDNA *miR-200c* were treated with DMEM supplemented with 100 μM β-glycerophosphate and 10 μM ascorbic acid. After 7 and 14 days, transcript and protein levels of odontogenic differentiation markers, including Runx2, OCN, DMP1, and DSPP, were analyzed using qRT-PCR and Western blot. The antibodies for DMP1, DSPP, and OCN were purchased commercially (R&D Systems, Minneapolis, MN, USA).

To determine the anti-inflammatory function of *miR-200c* overexpression, primary human DPCs were treated with *Pg*-LPS at 0.1 μg/mL after transfection with 0.1 μg pDNA *miR-200c* or EV facilitated by PEI. The transcripts of proinflammatory *IL-6* and *IL-8* were quantified using qRT-PCR.

### 4.3. Determining the Functions of pDNA miR-200c Delivered by CaCO_3_-Based Nanoparticles on Odontogenic Differentiation and Anti-Inflammation in Human DPSCs

The nanocomplexes of CaCO_3_/pDNA *miR-200c* or EV were prepared at different ratios of CaCO_3_ and co-precipitated protamine sulfate (PS) as previously described [40]. The average size and Zeta potential of nanocomplexes were measured using a scanning electron microscope (Hitachi 4800S, Tokyo, Japan) and Zetasizer Nano ZS (Malvern Instruments, Malvern, UK), respectively. To determine the influence of the ratio of CaCO_3_/PS on *miR-200c* transfection efficiency, 10^5^ DPSCs were treated with 1 µg pDNA *miR-200c*/CaCO_3_ nanocomplexes with CaCO_3_/PS ratio of 1:0.03125, 1:0.125, 1:0.25, and 1:0.5. The *miR-200c* expression was quantified and compared to other delivery systems, including naked pDNA and PEI delivery system after three days. In addition, the *IL-6* transcript was measured to determine the biocompatibility of CaCO_3_/pDNA nanocomplexes. The functions of CaCO_3_/miR-200c nanocomplexes on anti-inflammation and odontogenic differentiation of DPSCs were investigated by measuring *IL-6* transcripts under *Pg*-LPS challenge, and odontogenic differentiation markers, including OCN, DMP1, and DSPP, under odontogenic support medium after different time points. The proteins of odontogenic markers and the mineralization of DPSCs were analyzed using Western blot and Alizarin red staining after 14 days.

### 4.4. Determining the Functions of pDNA Encoding miR-200c in Rat Models of Pulpitis

All in vivo animal experiments were performed under the approval of the Office of Animal Resources at our university (#9012195), and all animal surgeries were performed under sterile conditions. A rat model of pulpitis was created by local application of *Pg*-LPS according to previous studies [49]. Briefly, after anesthesia of eight-week-old Sprague Dawley (SD) pathogen-free rats (Charles River Laboratories, Wilmington, MA), Class I cavities of 0.5 mm depth were prepared on the mesial pit of occlusal surfaces on maxillary first molars using ¼ round carbide burs. A pinpoint size of the pulp was exposed using the tip of an endodontic explorer, and the bleeding was controlled using a paper point (Dentsply Sirona). To determine the pulpal inflammation induced by *Pg*-LPS and the anti-inflammatory function of pDNA *miR-200c*, after 10 μg *Pg*-LPS suspended in 1 μL PBS was added to the exposed pulps, a collagen sponge (1 × 1 × 1 mm^3^) incorporated with either naked EV (1 μg) or pDNA *miR-200c* (1 μg) was implanted. All cavities were subsequently filled with Fuji Triage (GC) (Figure 5A). The anti-inflammatory function of *miR-200c* in pulp inflammation was determined after seven days using histological examination.

To determine if *miR-200c* promotes dentinogenesis, CaCO3/*miR-200c* nanocomplexes with CaCO3/PS ratio of 1:0.25 were prepared and subsequently incorporated into trimmed collagen sponges and lyophilized. After rat pulpitis was created at the maxillary first molars, the injured site was treated with collagen sponge incorporated with CaCO3/*miR-200c* at 0.1 μg. CaCO_3_/empty vector (EV) at the same dose was used as controls. Histological assessment of dentin repair with different treatments was performed after three weeks. There were 3 rats for each treatment.

### 4.5. Quantitative Reverse Transcription PCR

Total RNA was extracted using Qiagen miRNeasy microKit (Qiagen, Hilden, Germany). MirX (Takara, Kyoto, Japan) cDNA synthesis kits were used to detect miRNA expression. Quantitative PCR was performed using SYBR Premix Ex TaqTM II (Takara). Comparative real-time PCR was performed in replicate, and relative expression was obtained using the comparative Ct (ΔΔCt) method.

### 4.6. Western Blot

Cells with different treatments were isolated using RIPA buffer (Sigma) supplemented with protein inhibitor (Sigma). 20 μg of each sample was separated on an SDS-PAGE and transferred to nitrocellulose transfer membranes. Primary antibodies at different concentrations were incubated overnight and labeled using a secondary antibody (anti-mouse IgG conjugated with HRP, Promega, Madison, WI, USA) in 1:10,000 in a blocking buffer.

### 4.7. Histological Examination

After euthanasia, the rat maxilla was harvested and fixed in 4% paraformaldehyde for 24 h at 4 °C, followed by decalcification using 10% ethylenediamine-tetraacetate (EDTA) for three weeks. After decalcification, the samples were dehydrated in a gradient of ethanol before being immersed twice in xylene. All samples were then embedded in paraffin and sectioned. Sagittal sections, 7 μm thick, were cut perpendicular to the tooth axis before staining. Sections were stained with hematoxylin-eosin (H&E) and Heidenhain’s AZAN trichrome to visualize inflammation and reparative dentin. In the rat model of pulpitis, immunohistochemical (IHC) staining using an antibody of IL-6 was also performed. The histological examination was performed in double-blinded manner. To quantify the severity of pulpal inflammation mediated by *miR-200c,* we have scored the inflammatory cell response for pulpitis treated with CaCO3 delivered pDNA *miR-200c* and EV in a double-blinded manner according to previously published criteria [42].

### 4.8. Statistical Analysis

Descriptive statistics were conducted for both in vitro and in vivo investigations. A one-way analysis of variance (ANOVA) with post hoc Tukey’s honestly significant difference (HSD) test was used to determine whether there were significant differences between treatment groups for the in vitro *miR-200c* transfection, proinflammatory cytokines, and odontogenic markers. A Student’s *t*-test assessed differences in proinflammatory cytokines and *miR-200c* transcripts in human pulpitis. The Shapiro–Wilk test was also applied to verify the assumption of normality. All statistical tests completed for the in vitro and in vivo quantifications used a significance level of 0.05, and each graphic depicts mean values and associated standard deviations. Statistical analyses and associated figures were created via GraphPad Prism 10.3.1 (509).

## Data Availability

Data is contained within the article and Supplementary Material. The data presented in this study are available on request from the corresponding author.

## Supplementary Materials

The following supporting information can be downloaded at: https://www.mdpi.com/article/10.3390/ijms26146734/s1.
