# Supplementary material for: microRNA-200c Mitigates Pulpitis and Promotes Dentin Regeneration
Source: Int J Mol Sci. 2025 Jul 14;26(14):6734. doi: 10.3390/ijms26146734 (PMC12296186; doi:10.3390/ijms26146734)
Supplement: Supplementary file 1 [file ijms-26-06734-s001.zip › ijms-3698893-supplementary.pdf]

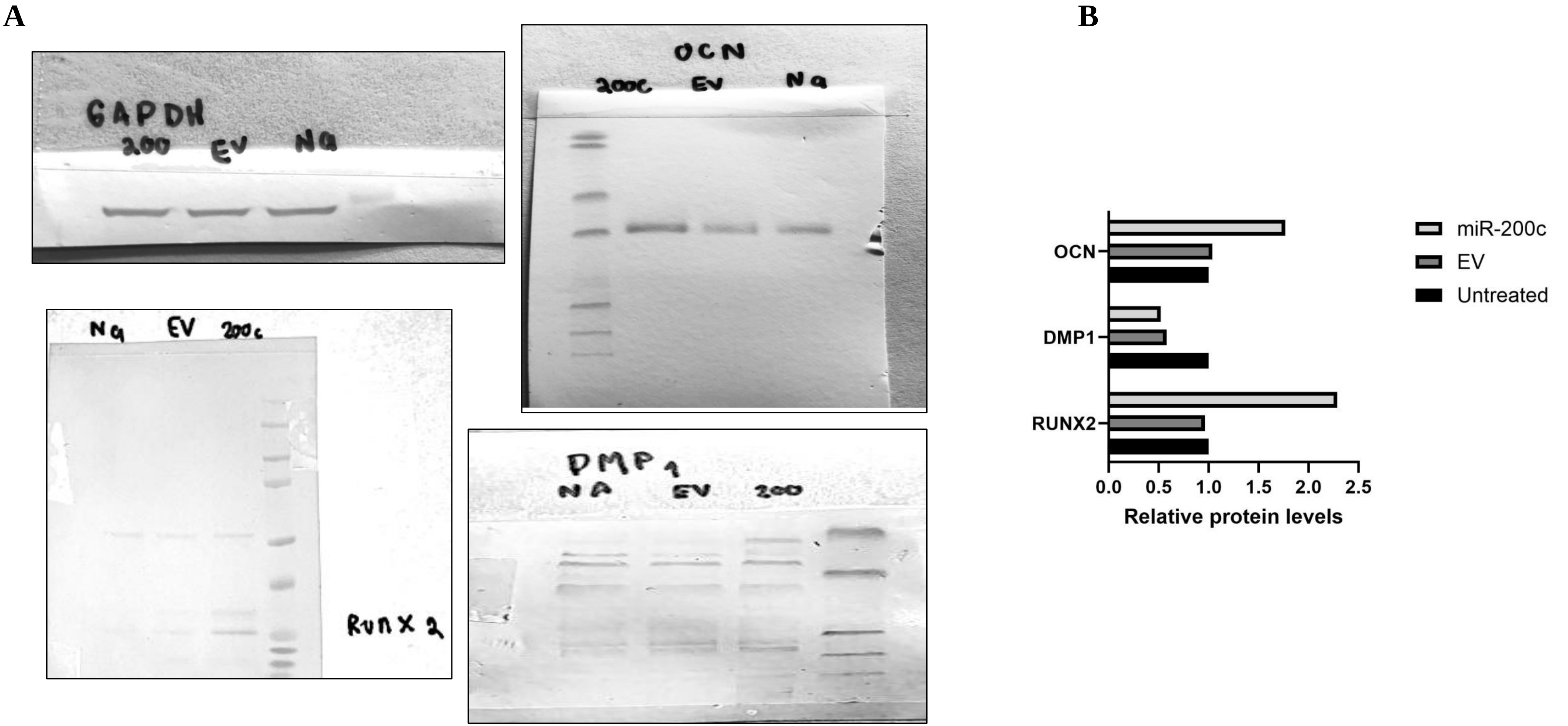


**Supplementary Figure S1: A:** Raw images of western blot of DMP1, OCN, and Runx2 in human DPSCs 14 days after treatment with 0.1 µg *miR-200c*, empty vector, and un-treated controls. **B:** Quantitative measurement of band intensities using ImageJ/Fiji 1. 2.3.0 [https://www.nature.com/articles/nmeth.2019] and results normalized to the untreated and loading control to obtain relative protein levels.

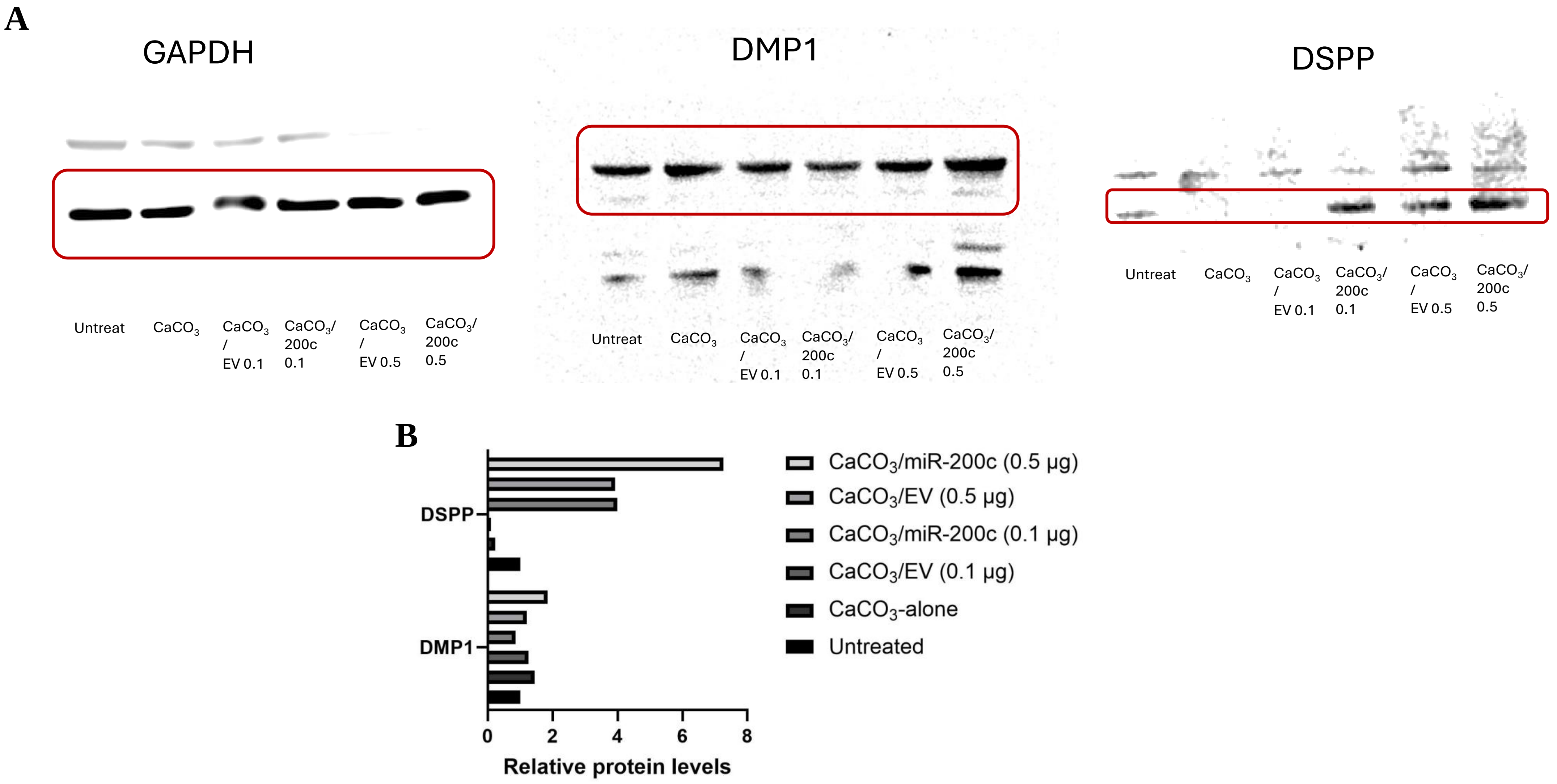


**Supplementary Figure S2: A**: The raw images of western blot for DMP1 of DPSCs with different treatments of CaCO3/ miR-200c. **B:** Quantitative measurement of band intensities using ImageJ/Fiji 1. 2.3.0 [https://www.nature.com/articles/nmeth.2019] and results normalized to the untreated and loading control to obtain relative protein levels.

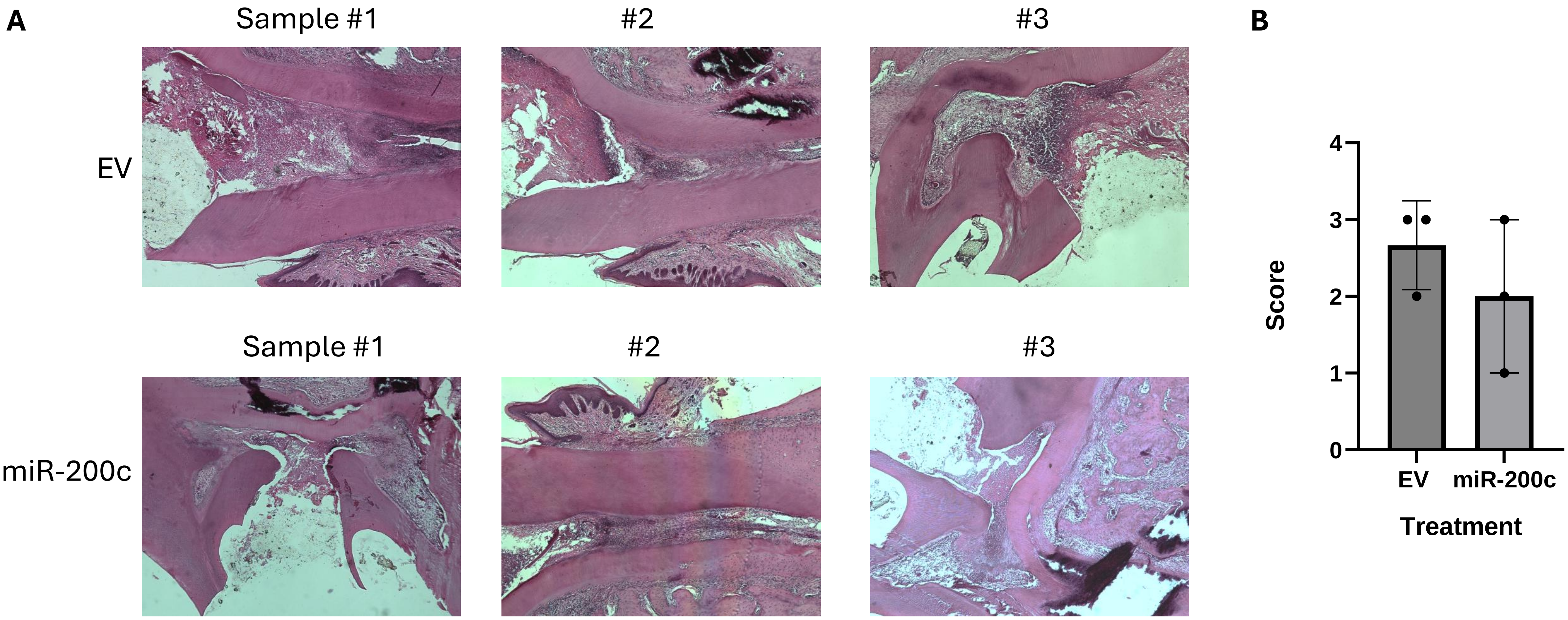


**Supplementary Figure S3:** The representative cross-section images (**A)** and the score of inflammatory cell response (**B**) of pulp tissue 3 weeks after treatment with collagen sponges incorporating CaCO3/pDNA miR-200c and CaCO3/EV at LPS-induced pulpitis of rats. H&E; Magnification: 4X; N=3
